# The effect of the first-generation HCV-protease inhibitors boceprevir and telaprevir and the relation to baseline NS3 resistance mutations in genotype 1: experience from a small Swedish cohort

**DOI:** 10.1080/03009734.2018.1441928

**Published:** 2018-03-14

**Authors:** Midori Kjellin, Terése Wesslén, Erik Löfblad, Johan Lennerstrand, Anders Lannergård

**Affiliations:** a Section of Clinical Microbiology, Department of Medical Sciences, Uppsala University Hospital, Sweden; b Section of Infectious Diseases, Department of Medical Sciences, Uppsala University Hospital, Sweden

**Keywords:** Boceprevir, hepatitis C, protease inhibitor, RAS, resistance association substitution, telaprevir

## Abstract

**Background:**

The clinical experience with protease-inhibitor (PI) triple regimen appears disappointing regarding effect, side effects, high work load, and costs. This real-world study evaluates baseline and emerging resistance-associated substitutions (RASs) and their significance for treatment outcome.

**Method:**

Thirty-six genotype 1a/b patients treated according to Swedish recommendations during 2011–2013 with triple therapy including pegylated interferon and ribavirin in combination with a protease-inhibitor, either boceprevir (BOC) or telaprevir (TVR), were retrospectively evaluated. Frozen serum samples from the patients were tested for resistance with pan-genotypic population sequencing.

**Results:**

Overall, 56% (20/36) of the patients achieved sustained viral response (SVR). The SVR was comparable between BOC (64%; 9/14) and TVR (50%; 11/22) (*p* = 0.07), and the IL28B type non-CC (48%; 12/25) and CC (46%; 6/13) (*p* = 0.77). The SVR was higher in patients without cirrhosis (89.5%; 17/19) (*p* < 0.0005), in treatment-naïve patients (70%; 14/20) (*p* = 0.02), and those with low viral load (<800,000 IU/mL) (66.7%; 8/12) (*p* < 0.0002), compared to those with cirrhosis (17.6%; 3/17), treatment-experienced (37.5%; 6/16), and high viral load (>800,000 IU/mL) (50%; 12/24).

**Conclusion:**

PI triple regimes were highly effective in treatment-naïve patients without cirrhosis, but in this real-world cohort an inferior effect was evident in cirrhotic and treatment-experienced patients. Although tested on a limited sample, the baseline resistance testing seems to have no impact on prediction of therapy outcome. The reason could be that the baseline RASs T54S and V55A have relatively low resistance towards BOC and TVR. Emerging RASs, mainly R155K, with known high resistance to BOC and TVR were frequently found in non-responders.

## Introduction

The global infection with hepatitis C virus (HCV) is estimated to afflict about 130 to 170 million people, who consequently have an elevated risk for liver cirrhosis and hepatocellular carcinoma (1–4). In Sweden, 45,000 patients with HCV are recorded by The Public Health Agency of Sweden (www.folkhalsomyndigheten.se), corresponding to a prevalence of HCV infection of about 0.4% (5). The genotypes (GT) 1 and 3, i.e. subtypes 1a, 1b, and 3a, are predominant in Sweden as in many other Western countries (6,7).

At the millennium 2000 the standard of care (SOC) therapy was introduced. This treatment was based on pegylated interferon (peg-IFN) and ribavirin (RIBA). The GT2 and 3 were found to be the easiest genotypes to treat, with an efficacy of up to 80%. However, for GT1 the efficacy was only up to 50% in patients with mild liver fibrosis and as low as 15%–20% in those with severe liver damage and liver cirrhosis (8).

As a result of the search for new targets in HCV therapy direct-acting antiviral (DAA) agents were developed to different HCV non-structural (NS) proteins such as NS3 protease, NS5A replication-associated protein, and NS5B polymerase. In the fall of 2011, the first generation of NS3 protease-inhibitors (PI), boceprevir (BOC) and telaprevir (TVR), targeting GT1, was approved in Sweden. These DAAs were used together with SOC (PI-triple), a combination that yielded a sustained viral response (SVR) at the level of 67%–75% in treatment-naïve mainly non-cirrhotic patients (9–12), at 69%–88% in prior relapse patients, and at 40%–52% in previous non-responding re-treated patients (13,14). In treatment-experienced patients with compensated cirrhosis Child A, the SVR was 39%–46% (15). In a real-world setting, it was shown that the treatment effect with BOC or TVR tended to be lower for cirrhotic patients, 38.9% compared to 65% for non-cirrhotic patients (16). Moreover, it was shown that host factors such as cirrhosis, ethnicity, albumin level, viral load ≥800,000 IU/L, and BMI ≥30 were significant predictors for response in a multivariate logistic regression analysis (16). Other studies supported these findings and also added age, gender, stage of fibrosis, and previous treatment experience to the list (17).

In 2009, it became evident that single nucleotide polymorphisms (SNP) located near the interferon-associated human gene, IL28B, had a high predictive value for the outcome of the GT1 SOC treatment response (18). In the rs12979860 location, the alleles could be occupied by either cytosine (C) or thymine (T). The most favorable treatment outcome was obtained with SNPs, in decreasing order, CC, CT, and TT (19). Although a specific study was not carried out, there were robust data that showed a better outcome for the patients with CC SNP compared with non-CC also in the PI-triple setting (11,20,21).

Resistance-associated substitutions (RASs) are amino acid substitutions in the virus that confer reduced susceptibly or resistance to a given DAA (22). Naturally occurring resistant (virus) variants that carry these RASs emerge and disappear continuously in the HCV quasi-species due to lack of proofreading by its DNA polymerase and high replication rate. During pressure from any given DAA treatment, selection of such RASs may render a population of resistant variants predominant, leading to viral break-through or relapse (22). For BOC and TVR, the most potent resistance NS3-codon position is 155 (amino acid substitutions), and the R155K is the most commonly emerging RAS in GT1a treatment failures. This RAS can confer cross-resistance to other PIs such as today’s approved drugs simeprevir and paritaprevir (23,24). The GT1a infections are more difficult to treat with PIs as this subtype is more prone to develop resistance mutations than subtype 1b (25–27). The half-life of GT1a emerging NS3 RASs by BOC and TVR (usually R155K) is estimated to be 14 and 10.6 months, and for GT1b 12.5 and 0.9 months, respectively (28). Thus, the patients who fail PI treatment usually have emerging RASs with a half-life of one year, which could prohibit re-treatment with the same drug class during at least two years (26,28).

As indicated above, even treatment-naïve patients could have RASs against PIs, i.e. resistance at baseline. These baseline RASs/polymorphisms do not affect the replication capability of the virus significantly compared with the wild-type form, and therefore in some patients they can constitute the dominant virus variant (28). Baseline RASs in GT1a towards BOC/TVR were found in 8.9% of treated patients. The earlier studies did not point out the clinical significance of baseline RASs in the context of SVR, because many patients had a favorable outcome (29).

The population-based (Sanger) sequencing, which can detect single mutations down to the 20% level, is the most commonly used method in clinical studies. In prevalence investigations, different methods such as deep sequencing (detection limit down to 1%) can be used (27,30). However, the general consensus is to recommend a cut-off level of 10%–20%, for detecting RASs within the HCV quasi-species, in order to be of clinical relevance in predicting viral failures (22).

Side effects with the PI-triple treatment were more pronounced than with SOC only, and the efforts to mitigate these effects were strenuous to the health professionals (31). Later, from 2014 in Sweden, SOC was generally not recommended in the treatment arsenal, partly depending on the frequent, often severe side effects, waiting for more effective and more non-toxic DAAs to be developed. BOC and TVR were only used until 2013. The approval of more potent DAAs in combination against NS3, NS5A, and NS5B has since 2014 reduced the duration of the treatment and increased the cure rate to more than 90% for all GTs.

The aim of this study was to investigate the effect of the PI-triple treatment in our patient group and to relate the outcome to baseline NS3 resistance. We also wanted to register adverse events and working load.

## Patients and methods

This retrospective, single-center, real-world study was performed from October 2011 to May 2013. Included patients received treatment with the PI-triple at the Department of Infectious Diseases, Uppsala University Hospital. The study was approved by the Regional Research Ethics Committee in Uppsala (Dnr 2013/185). The inclusion criteria were: Infection with GT1a and 1b; ≥18 years of age; treatment according to Swedish consensus recommendations 2012 (32); treatment with at least one dose of the treatment regime; informed consent was obtained. Child–Pugh score was approximated from the level of liver elasticity (33), biochemical results, or ultrasound. Data were captured from the patient records. For determining pre-existing RAS and IL28B (rs12979860), stored, frozen (minus 20 °C at the Uppsala Biobank) blood samples were used. At the time of the retrospective analysis, it became apparent that 5.8% (3/52) of the samples were missing in the biobank. SVR was regarded as HCV-RNA below lower limit of quantification (LLOQ) at 12 and 24 weeks after end of treatment (SVR12 and SVR24, respectively). Non-SVR was regarded as: viral break-through (a positive viral load nadir followed by a higher level); viral relapse (non-detectable viral load at the end of treatment followed by a higher level and finally); mortality before SVR24.

As an indirect measure on adverse events and work load, we used the number of visits and contacts with both doctors and nurses that were registered in the patient’s notes during the treatment. In case of more than one medical entry during one day, the entries were counted as one contact. At each of the visits to a nurse predetermined blood tests were done, and the patients were asked if there were any side effects since last visit. As for comparable use, we made the choice to use the regular treatment protocol for SOC, in which there was a predefined number of visits and, if rapid viral response (RVR), the treatment duration was determined to 24 weeks, otherwise 48 weeks.

### Laboratory methods

HCV-RNA quantification and resistance analyses of RAS (baseline and emerging) were performed at the Department of Clinical Microbiology at Uppsala University Hospital. The HCV-RNA level was analyzed using Roche COBAS^®^ AmpliPrep/TaqMan^®^ HCV Quantitative Test, v2.0 with a LOQ of 15 IU/mL. For NS3-resistance analyses of RASs, a nested PCR method was adopted, followed by Sanger sequencing (population sequencing). The mutations observed from the sequence analyses were compared with reported mutations. The pan-genotypic NS3 resistance method for RNA extraction, reverse transcription, nested PCR, and sequencing have been described elsewhere (34). In brief, RNA extraction from the samples was done using BioMérieux NucliSENS^®^ easyMAG™ system. cDNA was synthesized from RNA template with SuperScript™ III Reverse Transcriptase (Invitrogen™, Thermo Fisher) using random hexamers. First round PCR and nested PCR were performed with in-house primers targeting parts of the NS3-region using the TaqPCR Master Mix (QIAgen) (34). The integrity of the nested PCR products was verified by agarose-gel electrophoresis. PCR-positive samples were purified using QIAquick^®^ PCR Purification Kits. All protocols used were performed according to the manufacturer’s instructions. The purified products were sent to Uppsala Genome Center for capillary electrophoresis (Sanger) sequencing using the same primers as in the nested PCR. The HCV NS3 sequences were analyzed using SeqScape^®^ Software v2.6. The NS3 sequence of genotype 1a H77 strain was used as a reference template. The mutations were interpreted as relevant NS3 RASs by comparing with reported RASs (35,36). Thus, mutations/polymorphisms considered as potential RASs in this study are found at codon positions 36, 54, 55, 80, 122, 155, 156, 168, and 170.

Additional analyses on IL28B were performed at the Department of Clinical Pharmacology, Uppsala University Hospital. The methods for DNA extraction and sequencing have been described elsewhere (18). The liver elasticity (kPa) was measured with Fibroscan^®^ 502 by experienced nurses or doctors, approved by Echosens™.

### Statistics

The basic statistical computing was done in Microsoft^®^ Excel^®^ 2013 (Microsoft Office professional plus 2013, Microsoft Corporation). The Mann–Whitney non-parametric *U* test was used to test the differences between two groups with continuous data. The data were computed by PAST 3.11 (Øyvind Hammer, February 2016; Hammer Ø, Harper DAT, Ryan PD, 2001). A *p* value of <0.05 was regarded statistically significant.

## Results

None of the identified 36 patients were excluded due to unwillingness to participate. In total, 55.5% (20/36) were treatment-naïve (N), 16.7% (6/36) prior relapsers (RR), 13.9% (5/36) partial responders (PR), 11.1% (4/36) null responders (NR), and one patient discontinued prematurely due to pulmonary embolism. The distribution of treatment experience between the SVR and non-SVR groups was comparable, as were the body mass index (BMI) and gender. The SVR group was younger than the non-SVR group, 47.7 versus 55.9 years of age (*p* = 0.03). The proportion of patients with liver cirrhosis (Child A and B score) was lower in the SVR group compared with the non-SVR group, 7/20 and 15/16, respectively (*p* = 0.02), and the same was the case for liver elasticity, with mean values of 9.5 and 15.3 kPa, respectively (*p* = 0.02).

### Efficacy

Overall, 56% (20/36) of the patients achieved SVR. The SVR was comparable between BOC (64%; 9/14) and TVR (50%; 11/22) (*p* = 0.07), and the IL28B type non-CC (48%; 12/25) and CC (46%; 6/13) (*p* = 0.77). The SVR was higher in patients without cirrhosis (89.5%; 17/19) (*p* < 0.0005), in treatment-naïve patients (70%; 14/20) (*p* = 0.02), and in those with low viral load (<800,000 IU/mL) (66.7%; 8/12) (*p* < 0.0002), compared with those with cirrhosis (17.6%; 3/17), treatment-experienced (37.5%; 6/16), and high viral load (>800,000 IU/mL) (50%; 12/24). Of the non-responders, 9/16 had a viral break-through, 6/16 a relapse, and one patient died before SVR24 (see the adverse events section).

### Adverse events

The mean number of contacts with nurses was higher than expected in the ordinary treatment protocol (SOC), 20.4 (SD ±10.9) versus 11.3 (SD ±3.0). The mean number of contacts with doctors per patient (including phone calls and administrative duties documented in the patient’s notes) during treatment was 11.5 (SD ±1.2), which was comparable with the regular SOC schedule (data not shown).

Anemia was common. The serum hemoglobin concentration decreased with at least 26 g/L in all patients, with a mean drop of 49 (SD ±13.5) g/L. There was no difference between the BOC or TVR groups. Measures to deal with the anemia were: expectancy (*n =* 15); RIBA dose reduction only (*n =* 12); RIBA dose reduction, erythropoietin treatment, and blood transfusions (*n =* 4); RIBA dose reduction and blood transfusions (*n =* 3); and RIBA dose reduction, erythropoietin treatment (*n =* 1). One patient suffered from hemolytic anemia and consequently was treated with prednisolone as well as RIBA dose reduction. Measures to deal with the reduction of hemoglobin levels were comparable between the SVR and non-SVR groups.

Eight patients discontinued the treatment. The cause was autoimmune hemolysis, septic shock, or worsened skin psoriasis in the BOC group (*n =* 3). The cause for discontinuation in the TVR group was severe rash which covered more than 50% of the body surface in four patients, and another patient died from massive pulmonary embolism secondary to liver cancer.

### Baseline and emerging RASs

Presence of baseline NS3 RASs was found in both the SVR and non-SVR groups. Missing data was apparent in two patients in the SVR group and four in the non-SVR group. In total, 11% (4/36) of the patients were found to have virus strains with baseline RASs, three patients in the SVR group and one in the non-SVR group. In the SVR group, T54S (*n =* 2) and V55A (*n =* 1) and in the non-SVR group T54S were found (Table 1). The strain in the non-SVR group exhibited the same T54S as the emerging RAS after TVR-treatment (Table 1). Interestingly, two patients in the SVR group had HCV strains with Q80K and one patient with D168G (Table 1).

**Table 1.** Baseline RASs in the SVR-group and the non-SVR-group.

	Patient	Genotype	Subtype	RAS	Natural prevalence	Mean fold change in resistance compared to wild-type replicon
The SVR group	7	1	a			
	8	1	a			
	11	1	a	T54S	0.4%–3.1%	2.0–20.0
	15	1	a			
	16	1	a			
	18	1	a	T54S	0.4%–3.1%	2.0–20.0
	20	1	a	Q80K^a^	4.8%–75.0%^a^	NA^a^
	26	1	a	Q80K^a^	4.8%–75.0%^a^	NA^a^
	27	1	a	D168G^a^	NO^a^	2.0–20.0^a^
	29	1	a			
	30	1	a			
	35	1	a	T55A	2.8%	2.0–20.0
	9	1	b			
	19	1	b			
	25	1	b			
	31	1	b			
	32	1	b			
	34	1	b			
	17	1	Missing			
	22	1	Missing			
The non-SVR group	2	1	a			
	3	1	a			
	4	1	a			
	5	1	a			
	6	1	a			
	10	1	a			
	13	1	a	T54S	0.4%–3.1%	2.0–20.0
	14	1	a			
	23	1	a			
	24	1	a			
	28	1	a			
	33	1	a			
	36	1	a			
	1	1	a			
	12	1	b			
	21	1	b			

a Q80K and D168G are not representative RASs for BOC or TVR.

Emerging RASs were detected in 57.1% (8/14, two samples missing). At viral break-through, 62.5% (5/8) of the patients had selected strains with emerging RASs: one with R155K and four with V36M/R155K. At viral relapse, 50% (3/6) of the patients had emerging RASs: one with T54S/R155K, one with T54S, and one with V36M. Five patients had no detectable RAS (Table 2).

**Table 2.** Emerging representative RASs during and after treatment with BOC or TVR, mainly V36S and R155K, in 57.1% of the non-responders.

Patient	Genotype	Subtype	RAS	Natural prevalence	Mean fold change in resistance compared to wild-type replicon	Treatment response
2	1	a	R155K	0.2%–0.9%	2–100	VB
3	1	a	Negative			VB
4	1	a	V36M, R155K	0.2%–0.6%; 0.2%–0.9%	2–20; 2–100	VB
5	1	a	V36M, R155K	0.2%–0.6%; 0.2%–0.9%	2–20; 2–100	VB
6	1	a	V36M, R155K	0.2%–0.6%; 0.2%–0.9%	2–20; 2–100	VB
10	1	a	T54S, R155K	0.4%–3.1%; 0.2%–0.9%	2–20; 2–100	VR
13	1	a	54S	0.4%–3.1%	2.0–20.0	VR
14	1	a	V36M	0.2%–0.6%	2.0–20.0	VR
23	1	a	Negative			VR
24	1	a	R155K	0.2%–0.9%	2–100	VB
28	1	a	Negative			VB
33	1	a	V36M, R155K	0.2%–0.6%; 0.2%–0.9%	2–20; 2–100	VB
36	1	a	Negative			VR
1	1	a	Missing			VB
12	1	b	Negative			VR
21	1	b	ND			AM

AM: *ad mortem* before end point; VB: viral break-through, a positive or negative viral load nadir, followed by a higher level; VR: viral relapse, a non-detectable viral load at the end of treatment, followed by a higher level.

## Discussion

One of the purposes of this retrospective study was to study outcome in our patient population. The study showed that the efficacy of the PI-triple concept (SVR 70%) in treatment-naïve patients was comparable with the manufacturer’s registry studies performed before the approval of the first-generation protease inhibitors BOC and TVR. In non-cirrhotic patients the SVR was as high as 89.5%. However, in cirrhotic and treatment-experienced patients the SVR only reached 17.6 and 37.5%, respectively, which was lower than reported in the registry studies (9–14). Thus, the real-world data show similar SVR in treatment-naïve and non-cirrhotic patients as the registry studies but lower SVR in cirrhotic and treatment-experienced patients. The reason for this discrepancy cannot be fully explained but may have a basis in a high degree of co-morbidities. The combination of a relatively weak PI and cirrhosis, Child–Pugh A, in nearly all patients in the non-SVR group would be another cause for the many failures and may predispose for the selection of resistance strains. Emerging RASs with some or high clinical significance was found in 64% (9/14) of available serum from non-responders (14/16). SVR was higher in patients with low viral load, but age, gender, BMI, or IL28B had no impact.

The population-based (Sanger) sequencing was used to evaluate the existence of baseline RASs. Only occasional cases of RASs with impact on BOC and TVR treatment were found. These RASs consisted of substitutions at T54S and V55A with a natural prevalence of approximately 3% (24). The mean fold change in resistance to BOC and TVR for these RASs compared with wild-type is 2–20 and may be of minor importance. Three out of four patients with these RASs were successfully treated, which could be explained by the relatively low resistance towards BOC and TVR of the baseline RASs T54S and V55A (28). One patient still harbored the same T54S RAS after viral relapse. This specific patient suffered from a SOC-induced autoimmune hemolysis, liver cirrhosis (Child A), had a previous relapse after SOC, and had a high viral load at treatment start. During treatment he reached a RVR and continued to be virus-negative during the treatment course of 22 weeks.

Noteworthily, baseline RAS Q80K was found in two and D168G in one patient with GT1a in the SVR group. These RASs have no importance for the first-generation PI. It should be noted that Q80K has specific *in vitro* resistance towards simeprevir of approx. 10-fold change in resistance compared with wild-type replicon (35,36). The Q80K has a high prevalence in HCV genotype 1a strains, 47% in the United States (37), 19.8% in Europe, and 15.2% in Sweden (24). In genotype 1b, this RAS is rare (24). In our limited study the Q80K for genotype 1a was detected with a prevalence of 5.5%. It was shown, in a subanalysis from a trial with simeprevir and sofosbuvir, that the presence of Q80K contributed to a 12% relapse rate compared to 4% without (38). The prevalence of D168G is low in GT1a (39) but may contribute to a lower effect of simeprevir-containing treatment combinations (40).

The SVR rate among patients with cirrhosis is lower than was first reported from the studies that formed the base for registrations. These studies were mainly powered to show non-inferiority to SOC. Subgroup analyses on cirrhosis patients were performed on small groups, mostly some tens of patients. These more descriptive observations on per protocol groups showed SVR rates between 20% and 80%, the latter with only 22 patients (9,11,13,22). Later real-world studies per protocol observations showed SVR rate up to 54% in compensated cirrhotic patients and 35% in decompensated. The dropout rates in these studies were as high as 40% (41,42), meaning that the ITT SVR rates, presumably, were considerably lower. Lastly, in our study, there was one dropout due to death, and 12 out of 17 were treated with TVR. The SVR rates in previous relapsers and naïve patients were 30% and 40%, respectively. Patients older than 65 years of age, previous null-responders, and partial responders did not achieve SVR.

This study is a retrospective and single-center study, and the results should therefore be interpreted with caution. For example, the adverse events could not be fully evaluated due to irregular documentation in the patient’s notes. The differences in working load at the clinic have been estimated from historical routine schedules. The study could have been improved if a matched control group treated with SOC had been included. The statistic calculations have been done only with univariate analyses. However, the presented data reflect the real-world conditions with the high clinical work load experienced, especially by the nursing staff. It is questionable, in the rearview perspective, if these PI-triple regimes with BOC or TVR should have been approved for patients with the most advanced liver diseases.

Lessons should have been learnt from the HIV era with the rapid approval of drugs in the 1990s, for example stavudine (d4T) and didanosine, which are no longer in use in HIV treatment due to their severe adverse effects (43).

## Conclusion

PI-triple regimes were highly effective only in treatment of HCV GT1, in naïve patients without cirrhosis, and not in patients with cirrhosis and/or the treatment-experienced. Baseline IL28B and resistance testing with population sequencing in this limited study seem to have no impact on predicting therapy outcome. One reason could be that the baseline polymorphism/RASs T54S and V55A have relatively low resistance towards BOC and TVR. However, emerging RASs, mainly R155K, with known high resistance to BOC, TVR, simeprevir, and paritaprevir were frequently found in non-responders. It is important to detect such RASs in order to predict re-treatment outcome with the currently used PIs, for example paritaprevir.
